# Multidrug resistant pathogens respond differently to the presence of co-pathogen, commensal, probiotic and host cells

**DOI:** 10.1038/s41598-018-26738-1

**Published:** 2018-06-05

**Authors:** Agnes P. Chan, Yongwook Choi, Lauren M. Brinkac, Radha Krishnakumar, Jessica DePew, Maria Kim, Mary K. Hinkle, Emil P. Lesho, Derrick E. Fouts

**Affiliations:** 1 J. Craig Venter Institute (JCVI), 9605 Medical Center Drive, Suite 150, Rockville, MD 20850 United States; 2 Multidrug-resistant organism Repository and Surveillance Network, Bacterial Diseases Branch, Walter Reed Army Institute of Research, Silver Spring, MD 20910 United States; 3 Present Address: Infectious Diseases Unit, Rochester Regional Health, Rochester, NY 14621 United States

## Abstract

In light of the ongoing antimicrobial resistance crisis, there is a need to understand the role of co-pathogens, commensals, and the local microbiome in modulating virulence and antibiotic resistance. To identify possible interactions that influence the expression of virulence or survival mechanisms in both the multidrug-resistant organisms (MDROs) and human host cells, unique cohorts of clinical isolates were selected for whole genome sequencing with enhanced assembly and full annotation, pairwise co-culturing, and transcriptome profiling. The MDROs were co-cultured in pairwise combinations either with: (1) another MDRO, (2) skin commensals (*Staphylococcus epidermidis* and *Corynebacterium jeikeium*), (3) the common probiotic *Lactobacillus reuteri*, and (4) human fibroblasts. RNA-Seq analysis showed distinct regulation of virulence and antimicrobial resistance gene responses across different combinations of MDROs, commensals, and human cells. Co-culture assays demonstrated that microbial interactions can modulate gene responses of both the target and pathogen/commensal species, and that the responses are specific to the identity of the pathogen/commensal species. In summary, bacteria have mechanisms to distinguish between friends, foe and host cells. These results provide foundational data and insight into the possibility of manipulating the local microbiome when treating complicated polymicrobial wound, intra-abdominal, or respiratory infections.

## Introduction

The emergence of Multiple Drug Resistant Organisms (MDROs) that exhibit resistance to at least three classes of antibiotics^1^, and the frequency of serious infections caused by MDROs combined with the lack of development and approval of new antibiotics has led the World Health Organization (WHO), Centers for Disease Control and Prevention (CDC), and the Infectious Diseases Society of America (IDSA) to declare that antimicrobial resistance poses an urgent threat to global public health^2–4^. In addition to new antibiotics that are not inactivated by the resistance mechanisms described below^5^, alternative and or adjunctive treatment strategies are critically needed^6,7^.

*Klebsiella pneumoniae*, *Acinetobacter baumannii*, and *Enterobacter* species are some of the most challenging MDROs to treat, especially when they are isolated with other co-pathogens and/or commensals from polymicrobial respiratory, intra-abdominal and wound infections^8–10^. These organisms have evolved and/or horizontally acquired a diverse arsenal of resistance mechanisms^11^, including transmembrane transporters known as efflux pumps, drug neutralizing enzymes such the carbapenemases^12^ and extended spectrum beta-lactamases^13,14^, and mutations in ribosomal RNA (rRNA)^15^ or topoisomerase genes^16^ to escape the target of the drug. Plasmids^17^, integrative conjugative elements (ICE)^18^, integrons^19^, resistance islands^20^ and even bacteriophage^21^ have all aided and abetted in disseminating antibiotic resistance genes between diverse bacterial species. Despite all we know regarding resistance mechanisms, it is not clear what role, if any, co-infecting pathogenic, resident microbiota, or the human host may have in influencing the expression of antibiotic resistance genes and virulence factors and ultimately pathogenicity of infectious species.

Microbial communities consist of multiple species in commensal or symbiotic coexistence where cells can communicate via quorum sensing and influence interactions with each other. To detect these biological interactions, classical studies have used co-culture assays to monitor the effects of a pathogen/commensal species on the growth or survival of the target species. Such co-cultures have been implicated in the induction of genes and cellular structures required for competitive viability^22,23^. In a few cases, genome-wide transcriptomic changes have been measured and results from these studies suggest that microbial gene expression can be extensively reprogrammed by other microbes in the same niche^22,24–26^. Growth inhibition as a result of co-culture assays have been reported among human resident or pathogenic bacteria (such as *P*. *aeruginosa* against *S*. *aureus*^25^, and *Streptococcus sanguinis* against *Streptococcus mutans*^27^, and certain groups of soil bacteria and fungi (such as *Bacillus subtilis* against *Streptomyces coelicolor*^28^, *Collimonas fungivorans* against *Aspergillus niger*^26^, and *Pseudomonas fluorescens* against *Bacillus*, *Brevundimonas* or *Pedobacter*^22^. There are also examples demonstrating the influence of host factors on virulence strategies of the pathogen such as the induction of antibiotic resistance in the presence of human host cells^29,30^ or sub-inhibitory concentrations of antibiotics^31^. Co-culture studies are valuable in understanding co-infection with more than one type of MDRO^32^. For example, in some studies, co-colonization with carbapenem-resistant *Enterobacteriaceae* (CRE) and *Acinetobacter* has been associated with a higher level of resistance to carbapenems^33^. Co-infection can also manifest cross-kingdom effects by regulating the survival of the host. *Caenorhabditis elegans* showed better survival when co-infected with both *Candida albicans* and *A*. *baumannii* instead of *C*. *albicans* alone^34^. Thus, it appears that *A*. *baumannii* may attenuate the growth of a competing organism, thereby directly influencing the outcome of a co-infection and the survival of the nematode.

Although there have been microarray and RNA-sequencing (RNA-Seq) based transcription profiles of bacterial co-culture and host-pathogen interactions^22,35–38^, there is a lack of information on how different nosocomial MDROs respond to each other and to the inhabitants of the human skin microbiome, and whether this interaction might affect regulation of genes that ultimately influence the virulence capacity of the organisms. In this study, we conducted genome-wide RNA-Seq gene expression analysis to reveal the transcriptional regulation of individual MDROs when co-cultured with or confronted against another MDRO, selected probiotic and skin commensal organisms, or cultured human cells. The primary goal was to identify and characterize interactions among bacteria and host that influence the expression of virulence factors and antimicrobial resistance (AMR) in MDROs.

## Results and Discussion

### Whole Genome Sequencing of MDROs

MDROs used in this study were selected by collaborators at the Walter Reed Army Institute of Research (WRAIR) Multidrug-resistant Organism Repository and Surveillance Network (MRSN) as having one or more of the following characteristics: polytraumatic blast injuries, initial injuries in Afghanistan or Iraq, multi-drug resistant, isolated from traumatic wound surfaces or surveillance culture, and/or outbreak in the DOD health system from 2003–2015. Details of the multiple selection criteria that apply to each isolate are summarized in Supplementary Table S1. The antibiogram for the 3 selected MDROs was included as Supplementary Table S2. Bacteria considered human commensals and members of the human skin microbiome were chosen as confronting partners if they can be cultured aerobically at 37 °C and on media similar to the selected MDROs, and if their genome sequence was publicly available. Preference was also given to those isolates known to inhabit skin^39,40^. *L*. *reuteri* was chosen due to its growing use as a probiotic^41,42^.

Although strains of a given bacterial species are highly similar, their gene content can vary by as much as 35%^43^. The differences among bacterial strains tend to be mobile elements (e.g., prophage, plasmids, integrated elements) and genes encoding for O-antigen or capsular polysaccharides (CPS)^44–48^. These “flexible” regions tend to encode genes involved in cell surface structures (i.e., O-antigen, CPS, teichoic acid, S-layer, flagella, pili, and porins) as well as genes for resource utilization, including antibiotic resistance genes. These regions that vary between strains have been referred to as flexible genomic islands (fGIs)^49,50^. To avoid missing biologically important genes within MDROs that may not be present in generic reference genomes, and to more precisely map mRNA transcripts generated during bacterial co-culture experiments, draft genome sequencing of these clinically relevant MDRO isolates was conducted.

All three MDRO isolates were sequenced to a high depth of coverage (e.g., 79 to 115-fold) and improved using reads from other sequencing technologies, optical maps, and an in-house automated genome finishing tool, resulting in the genome finishing status of “Improved High-Quality Draft” (IHQD) (Table 1). Plasmid-related contigs were discovered in all three genomes: two in *A*. *baumannii* MRSN 7339, three in *E*. *hormaechei* MRSN 11489 and 23 in *K*. *pneumoniae* MRSN 1319. A list of the plasmid-related contigs can be found in Supplementary Table S3. In the *K*. *pneumoniae* and *E*. *hormaechei* isolates, plasmid-encoded genes were identified that belong to the Type IV secretion system, for example, the *tra* and *virB* gene clusters, likely involved in conjugation. For AMR genes, a total of 10 and 5 were annotated on plasmid-derived contigs in the *K*. *pneumoniae* and *E*. *hormaechei* isolates, respectively. The AMR genes encode for inhibitors against various drug class combinations of beta-lactams, aminoglycosides, quinolones, phenicols, sulfonamides, and/or trimethoprim. As for the *A*. *baumannii* isolate, interestingly, the AMR genes were not detected on the plasmid-derived contigs but instead 15 AMRs genes, against beta-lactams, aminoglycosides, quinolones, and trimethoprim, were identified on the chromosome. Plasmid-derived genes from the 3 MDROs can be found in Supplementary Tables S4–S6.

**Table 1:** Genomic features and metadata of bacterial genomes used in this study.

Repository ID	MRSN 1319	MRSN 7339	MRSN 11489	ATCC 55730	BEI HM-118	ATCC 43734
Organism	*K*. *pneumoniae*	*A*. *baumannii*	*E*. *hormaechei*	*L*. *reuteri SD2112*	*S*. *epidermidis SK135*	*C*. *jeikeium*
Accession	JSVB00000000	JPHV00000000	JTEP00000000	CP002844	ADEY00000000	ACYW00000000
Group	MDRO	MDRO	MDRO	Commensal	Commensal	Commensal
Origin	wound	wound	wound	breast milk	skin	blood culture
Sequencing Technology	Illumina HiSeq. 2000	Illumina HiSeq. 2000	Illumina HiSeq. 2000	Roche 454	Roche 454	Roche 454
Finishing Status^†^	IHQD	IHQD	IHQD	Finished	HQD	HQD
Average Coverage	79.23×	115.46×	94.99×	63×	26×	35×
#Contigs	52	34	28	5	33	93
Contig N50	392,725	246,634	332,009	na	na	na
Average Contig Length (bp)	105,180	116,337	165,823	463,368	76,304	26,091
Max. Contig Length (bp)	765,691	847,614	652,496	2,264,399	751,210	161,413
Total Genome size (bp)	5,469,353	3,955,466	4,643,032	2,316,838	2,518,045	2,426,461
Total G + C%	57.31	39.09	55.30	39.04	32.24	61.63
#Proteins	5,430	3,787	4,513	2,300	2,395	2,224
#Plasmid contigs	23	2	3	4	nd	nd
References	This study	Chan *et al*., 2015	This study	Spinler *et al*., 2014	BEI Resources	Jackman *et al*., 1987

^†^Improved High-Quality Draft (IHQD); High-Quality Draft (HQD).

na = Not available or applicable.

nd = Not determined.

### Co-cultures of selected MDROs and human skin microbiome species

The skin represents a complex microbial ecosystem, with interactions between microbial species, and between microbes and the host. Common skin inhabitants include bacteria, fungi, and viruses. Many of the microbes that colonize the skin are harmless and may play a beneficial role through colonization and protection against invasion by pathogens. The resident skin microbes may also offer an additional line of defense through priming the host immune system to counteract possible attacks by pathogens. The skin microflora consists of four major phyla: Actinobacteria (e.g., *Corynebacterium*, *Propionibacterium*, *Micrococcus*), Firmicutes (e.g., *Staphylococcus*), Bacteroidetes, and Proteobacteria^39^.

We selected *A*. *baumannii*, *K*. *pneumoniae*, *E*. *hormaechei* isolates from the WRAIR MRSN as the MDRO pathogen species, *S*. *epidermidis* and *Corynebacterium jeikeium* as the representative skin commensal residents, and *L*. *reuteri* as a probiotic representative, focusing on the responses of the MDROs to each other and the selected pathogen/commensal species. The pairwise co-culture assays among MDROs and the commensals or probiotics are summarized in Table 2.

**Table 2:** Bacteria-Bacteria Confrontation Assays.

Confronting bacteria		Commensal Controls	MDRO species		
			*E*. *hormaechei*	*K*. *pneumoniae*	*A*. *baumannii*
			EH	KP	AB
*L*. *reuteri*	**LR**	LR:LR	EH:LR	KP:LR	AB:LR
*S*. *epidermidis*	**SE**	SE:SE	EH:SE	KP:SE	AB:SE
*C*. *jeikeium*	**CJ**	CJ:CJ	EH:CJ	KP:CJ	AB:CJ
*E*. *hormaechei*	**EH**	na	EH:EH	KP:EH	AB:EH
*K*. *pneumoniae*	**KP**	na	na	KP:KP	AB:KP
*A*. *baumannii*	**AB**	na	na	na	AB:AB

na = Not available or applicable.

The total number of trimmed mapped reads from each organism per pairwise co-culture assay is summarized in Supplementary Table S7. A total of 388 and 321 *A*. *baumannii* genes (out of 3,860 genes in the genome) were up or down-regulated when co-cultured with *K*. *pneumoniae* and *E*. *hormaechei*, respectively. As many as 909 and 212 *K*. *pneumoniae* genes (out of 5,548 genes in the genome) were up or down-regulated when co-cultured with *A*. *baumannii* and *E*. *hormaechei*, respectively. Compared to the other two MDROs, *E*. *hormaechei* gene expression was only moderately affected when co-cultured with *A*. *baumannii* or *K*. *pneumoniae*, with 78 and 15 up or down-regulated genes out of 4,617 genes in the genome, respectively. A summary of differentially regulated gene counts obtained from co-cultures are summarized in Table 3.

**Table 3:** Differentially expressed gene in the MDROs *E*. *hormaechei*, *K*. *pneumoniae*, and *A*. *baumannii* when confronted with individual pathogenic/commensal bacteria.

MDRO species	#Annotated genes in genome	Regulation	Confronting Bacteria					
			*A*. *baumannii*	*K*. *pneumoniae*	*E*. *hormaechei*	*C*. *jeikeium*	*S*. *epidermidis*	*L*. *reuteri*
*A*. *baumannii*	3,860	Up	na	200	171	191	9	0
		Down	na	188	150	233	3	1
*K*. *pneumoniae*	5,548	Up	466	na	60	46	18	5
		Down	443	na	152	118	94	9
*E*. *hormaechei*	4,617	Up	71	13	na	0	4	0
		Down	7	2	na	0	0	1

Differentially expressed genes are defined as genes with an FDR <0.05 from edgeR analysis. Annotated genes included protein-coding, tRNA, tmRNA, and ncRNA.

na = Not available or applicable.

*S*. *epidermidis* (a skin resident) and *L*. *reuteri* (a common probiotic) stimulated the least gene expression responses when co-cultured with the MDROs. It is possible that the growth of commensal organisms was impaired by the MDROs in co-culture since growth inhibition has been observed previously in bacterial confrontations^22,23^. To determine if the MDROs were inhibiting the growth of the commensal organism, phenylethyl-alcohol and Hektoen Enteric selective agars were used to quantitate colony forming units of *S*. *epidermidis* and *E*. *hormaechei*, respectively during co-culture. No growth inhibition was observed (Supplementary Fig. S1), suggesting that slower growth patterns and low biomass at the end of the co-culture was the cause for the low amount of RNA-Seq reads, at least for the *E*. *hormaechei*-*S*. *epidermidis* confrontation. The same analysis could not be performed with the other commensal bacteria in this study because of their specific media requirements and the fact that other MDROs, *A*. *baumannii* and *K*. *pneumoniae*, both possess alcohol-dehydrogenase genes, which would have allowed growth on the selective phenylethyl-alcohol agar.

### Genes responsive in co-culture ranked by fold-change

We also examined extreme up/down gene responses of MDROs under co-culture conditions by requiring at least 4-fold up/down expression with FDR below 5%. For *A*. *baumannii*, a total of 25 and 19 genes were strongly up/down regulated respectively when co-cultured with *E*. *hormaechei* (Supplementary Fig. S2). A major fraction of the same gene set was similarly regulated when *A*. *baumannii* was co-cultured with another MDRO *K*. *pneumoniae*. Amongst the most strongly upregulated genes, by 11 to 12-fold, were alcohol dehydrogenase (T634_RS01410), aldehyde dehydrogenase (T634_RS01395), and lipoyl synthase (T634_RS06340). Two MFS transporters (T634_RS14350, T634_RS14800) and an outer membrane porin of the OprD family (T634_RS14805) were strongly downregulated by 5 to 10-fold.

As for *K*. *pneumoniae* gene responses, 24 and 14 genes were up/down regulated respectively when the co-culture was performed with *A*. *baumannii* (Supplementary Fig. S3). The strongest *K*. *pneumoniae* gene response was found in genes involved in iron acquisition, including TonB-dependent receptor (T643_RS07920; 45-fold), iron ABC transporter permease (T643_RS07935; 39-fold), hemin ABC transporter substrate-binding protein (T643_RS07930; 36-fold), and hemin-degrading factor (T643_RS07925; 34-fold). When the co-culture partner used was *E*. *hormaechei* instead, a different gene response was observed which involved a strong downregulation of the enterobactin gene clusters required for siderophore synthesis (discussed below).

Finally, a total of 25 *E*. *hormaechei* genes responded to co-culturing with *A*. *baumannii* or *K*. *pneumoniae*. Most genes were strongly upregulated when co-cultured with *A*. *baumannii* with the exception of a hypothetical protein (T636_RS15175) which was down-regulated (Supplementary Fig. S4). The most strongly upregulated *E*. *hormaechei* genes were involved in iron transport including ligand-gated channel protein (T636_RS04035), hemin uptake protein (T636_RS04030), hemin ABC transporter substrate-binding protein (T636_RS04045) by 21 to 32-fold when co-cultured with *A*. *baumannii*, followed by the enterobactin and aerobactin siderophore synthesis gene clusters (also discussed below).

### Regulation of AMR genes during co-culturing

**Figure 1:**
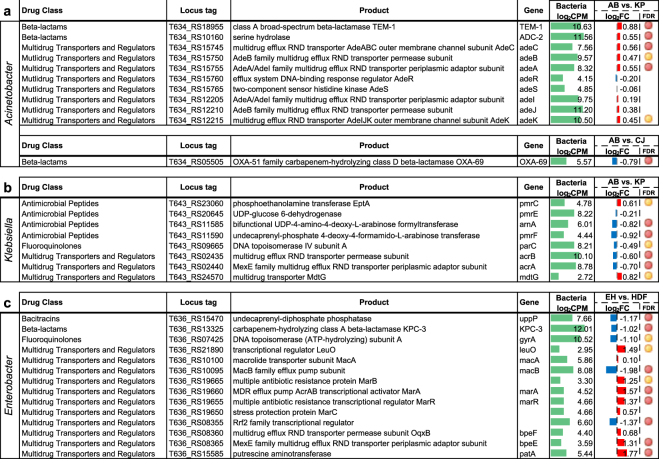
Antimicrobial resistance gene expression. Differentially regulated AMR genes in *A*. *baumannii* (AB) (**a**), *K*. *pneumoniae* (KP) (**b**) during bacterial co-culture experiments, and *E*. *hormaechei* (EH) in response to co-cultured HDF cells (**c**) are shown. **(**CJ) denotes C. *jeikeium*. Differentially regulated genes shown with edgeR false discovery rate (FDR): <0.05 (red circle) and <0.1 (yellow circle). Log_2_-fold change values of gene expression was shown as red (up-regulated) and blue (down-regulated) bar graphs with reference to monocultures. Bar scale from −4 to 4. Averaged gene expression level across control and treatment experiments (log_2_ read counts per million, log_2_CPM) was shown as green bar graphs. Bar scale from 0 to 15.

It is known that AMR is critically important to the survival strategy of the MDROs used in this study and that expression of AMR determinants has a fitness cost associated with it, but it is unknown to what extent AMR gene (ARG) expression is influenced by other co-cultured microbes. To determine effects of MDRO bacterial challenge on the expression of ARGs, we first identified ARGs using the Resistance Gene Identifier (RGI)^51^ in strict mode against the Comprehensive Antibiotic Resistance Database (CARD)^51–53^. RNA-Seq analysis showed significant differential expression of ARGs when *K*. *pneumoniae* and *A*. *baumannii* were co-cultured, with patterns of up- and down-regulation observed within each species (Fig. 1a,b). In *A*. *baumannii*, both the TEM-1 and ADC-2 beta-lactamase variants were upregulated when challenged with *K*. *pneumoniae* along with RND-family efflux systems *adeABC* and *adeIJK*. Conversely, in *K*. *pneumoniae*, lipid A modifying enzymes responsible for antimicrobial peptide resistance (*arnA*, *pmrE*, and *pmrF)*, multi-drug transporters *acrAB*, and quinolone resistance protein *parC*, were downregulated when challenged with *A*. *baumannii*. Exceptions of ARGs being up-regulated in *K*. *pneumoniae* were *pmrC* and *mdtG*. There were few significant ARG expression changes observed in MDROs when co-cultured with commensals. For *E*. *hormaechei*, no significant differentially expressed ARG was observed when challenged with any of the other bacteria – MDROs or commensals. One notable mention is the down regulation of the beta-lactamase OXA-69 (an OXA-51 subfamily carbapenemase^54^) in *A*. *baumannii* when co-cultured with the skin commensal *C*. *jeikeium* (Fig. 1a). This finding would be of considerable interest to the healthcare industry if the co-culturing of a skin commensal could result in an increase in susceptibility of *A*. *baumannii* to carbapenems through the lowering of the intrinsic OXA-51-like gene expression. This finding could also inform treatment decisions – for example by avoiding the concomitant use of vancomycin which kills commensals such as coagulase negative staphylococci and corynebacterial species.

When confronted with HDF cells, transcriptome analysis of *E*. *hormaechei* showed significant changes in ARG expression (Fig. 1c). Most notable, the antibiotic efflux complexes *marABC* and *bpeEF* were upregulated when challenged with HDF cells, while the *macB* periplasmic ATPase component of the *macAB* cassette and the KPC-3 beta-lactamase variant were downregulated. In *K*. *pneumoniae*, the aminoglycoside APH(3″)-Ib and beta-lactam resistance enzyme CTX-M-15 were downregulated in the presence of HDF. No significant differentially expressed ARGs were observed in *A*. *baumannii* when co-cultured with HDF cells.

### Differential expression of siderophore gene clusters

**Figure 2:**
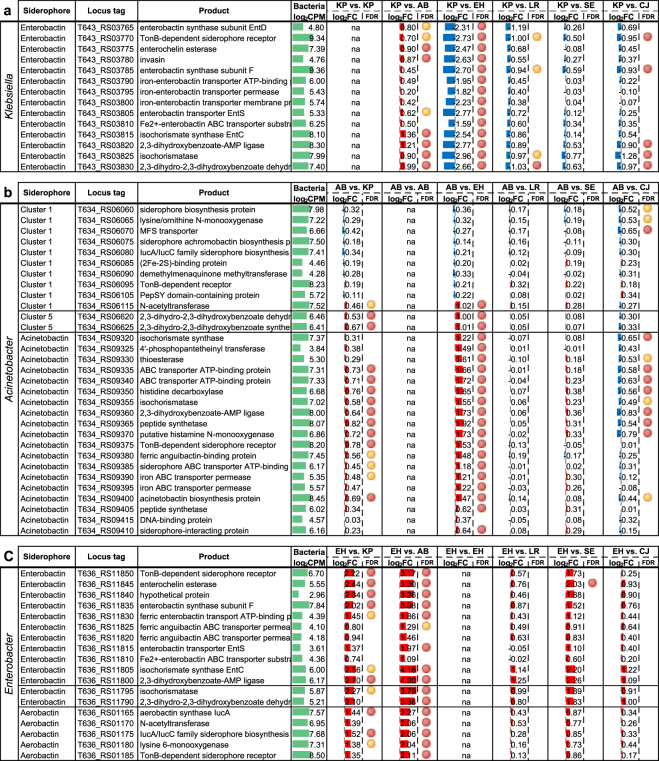
Siderophore gene cluster expression in (**a**) *K*. *pneumoniae* (enterobactin), (**b**) *A*. *baumannii* (acinetobactin), and (**c**) *E*. *hormaechei* (enterobactin-like and aerobactin) when co-cultured with individual pathogen/commensal species. *K*. *pneumoniae* (KP), *A*. *baumannii* (AB*)*, *E*. *hormaechei* (EH), *L*. *reuteri* (LR), *S*. *epidermidis* (SE) and *C*. *jeikeium* (CJ). Differentially regulated genes shown with edgeR false discovery rate (FDR): <0.05 (red circle) and <0.1 (yellow circle). Log_2_ fold change values of gene expression was shown as red (up-regulated) and blue (down-regulated) bar graphs with reference to monocultures. Bar scale from −4 to 4. Averaged gene expression level across control and treatment experiments (log_2_ read counts per million, log_2_CPM) was shown as green bar graphs. Bar scale from 0 to 15.

Siderophores are molecules essential for iron acquisition and the survival of bacteria, and are especially important for pathogens to compete for limiting free iron in an eukaryotic host environment. To determine how siderophore gene clusters are regulated during bacterial co-culture, we identified the corresponding siderophore gene clusters including acinetobactin in *A*. *baumannii*, enterobactin in *K*. *pneumoniae*, and both enterobactin-like and aerobactin in *E*. *hormaechei*. Genome-wide RNA-Seq expression and annotated virulence gene sets are shown in Supplementary Tables S4–S6. Across the 6 MDRO pairwise analysis performed, siderophore gene cluster expression was upregulated in almost all cases (Fig. 2). The only exception observed was when *K*. *pneumoniae* was co-cultured with *E*. *hormaechei* (i.e. KP vs. EH, Fig. 2a) in which case the *K*. *pneumoniae* enterobactin cluster was strongly downregulated by 8-fold (log_2_ fold change of 3) in the presence of *E*. *hormaechei*. Interestingly, although both *K*. *pneumoniae* and *E*. *hormaechei* encode enterobactin, only *K*. *pneumoniae* downregulated the transcription of enterobactin in the presence of *E*. *hormaechei* but not vice versa. Conceivably, *K*. *pneumoniae* may have evolved iron utilization feedback mechanisms to largely shut down production of enterobactin in favor of utilizing the enterobactin produced by *Enterobacter*. In contrast, the expression of the *A*. *baumannii* siderophore acinetobactin was consistently upregulated regardless whether the co-cultured pathogen was *K*. *pneumoniae* or *E*. *hormaechei*.

When the MDROs were individually co-cultured with the common skin resident *C*. *jeikeium*, a mild down-regulation of the *K*. *pneumoniae* and *A*. *baumannii* siderophore gene clusters was also observed (Fig. 2). The *C*. *jeikeium*-mediated reduction of siderophore production in competing co-cultured species could be a defense mechanism of commensals to outcompete invading pathogens in the human host.

### Differential expression of cell attachment components

**Figure 3:**
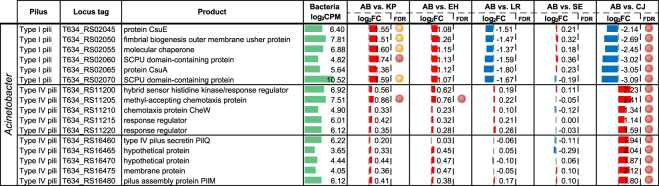
Regulation of *A*. *baumannii* type I and type IV pili gene cluster expression when co-cultured with individual pathogen/commensal species. *K*. *pneumoniae* (KP), *A*. *baumannii* (AB*)*, *E*. *hormaechei* (EH), *L*. *reuteri* (LR), *S*. *epidermidis* (SE) and *C*. *jeikeium* (CJ). Differentially regulated genes shown with edgeR false discovery rate (FDR): <0.05 (red circle) and <0.1 (yellow circle). Log_2_ fold change values of gene expression was shown as red (up-regulated) and blue (down-regulated) bar graphs with reference to monocultures. Bar scale from −4 to 4. Averaged gene expression level across control and treatment experiments (log_2_ read counts per million, log_2_CPM) was shown as green bar graphs. Bar scale from 0 to 15.

Pili and fimbriae systems are important for cell attachment and physical interactions; therefore, we also performed extensive annotation of the three selected MDRO genomes to identify genes involved in surface attachment, including type I and type IV pili and fimbriae proteins. RNA-Seq analysis showed that one of the three annotated *csu* chaperone gene clusters in *A*. *baumannii* (type I pili formation) was upregulated when co-cultured with *K*. *pneumoniae* by 3-fold, but distinctly downregulated almost 8-fold only when co-cultured with *C*. *jeikeium* (Fig. 3). This *csu* operon has been previously suggested to be involved in the adherence and formation of biofilm of *A*. *baumannii* on abiotic surfaces, but not human respiratory cells^55^. In addition, our transcriptome analysis also showed that two of the annotated type IV pili gene clusters in the *A*. *baumannii* strain were upregulated specifically when co-cultured with *C*. *jeikeium*. Type IV pili play a role in natural transformation, twitching motility, and biofilm formation^56^. Our results showed that *C*. *jeikeium* modifies type I and type IV pili expression of *A*. *baumannii*, which may be a mechanism to interfere with biofilm formation and surface attachment of the competitor species, in this case *A*. *baumannii*.

### Confirmation of gene expression by qPCR

RNA-Seq gene expression results were validated by performing qPCR for a select group of differentially expressed genes. Genes were chosen for analysis based on the ability to design unique primers and their reported fold change values. Biological replicates were tested and qPCR was performed on cDNA from reverse transcription of total RNA. For *K*. *pneumoniae*, 2 downregulated (*acnB*, T643_RS19850; *gabT*_2, T643_RS11485) and 3 upregulated (aminotransferase, T643_RS18930; *cimH*, T643_RS19470; PTS system fructose IIA component, T643_RS18905) genes were tested. For *A*. *baumannii*, 3 downregulated (*papD*, T634_RS02055; *mmsA*_1, T634_RS14320; EamA-like transporter, T634_RS10710) and 4 upregulated (*pilN*, T634_RS16475; NAD family protein, T634_RS01410; Hpt domain protein, T634_RS11200; hypothetical protein, T634_RS08500) genes were tested. Gene expression was normalized to *gyrB* (T634_RS06810, T643_RS05975) and relative expression ratios were determined by comparing co-culture samples with monoculture samples. Relative expression ratios were converted to log_2_FC and compared to the log_2_FC from RNA-Seq data. All genes tested showed the same direction of log_2_FC in both RNA-Seq and qPCR analysis (Supplementary Fig. S5), confirming the trends observed in the RNA-Seq analysis.

### Bacterial responses to human fibroblast co-culture assays

**Figure 4:**
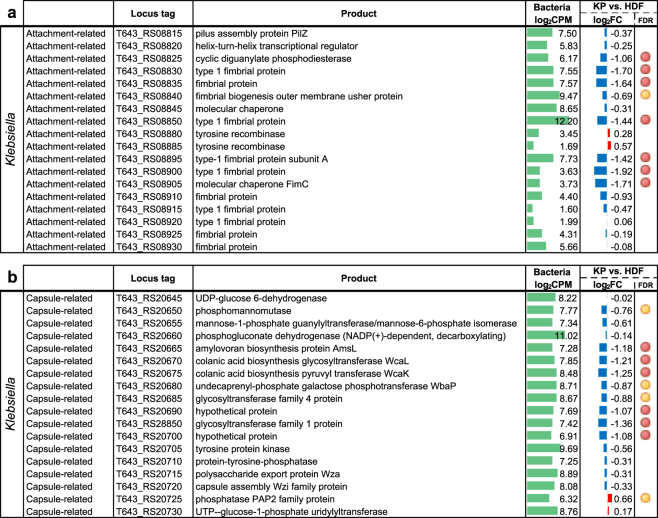
Expression of *K*. *pneumoniae* (**a**) attachment-related and (**b**) capsule-related gene clusters in response to co-cultured HDF cells. Differentially regulated genes shown with edgeR false discovery rate (FDR): <0.05 (red circle) and <0.1 (yellow circle). Log_2_ fold change values of gene expression was shown as red (up-regulated) and blue (down-regulated) bar graphs with reference to monocultures. Bar scale from −4 to 4. Averaged gene expression level across control and treatment experiments (log_2_ read counts per million, log_2_CPM) was shown as green bar graphs. Bar scale from 0 to 15.

To assess host interactions, each of the three selected MDROs for this study was also individually co-cultured with adult human fibroblast cells (HDF). Our transcriptome analysis using RNA-Seq showed that two gene clusters in *K*. *pneumoniae* were specifically down-regulated when co-cultured with HDF cells. The down-regulated *K*. *pneumoniae* gene clusters were involved in fimbriae production and capsule synthesis and showed a strong reduction of up to 3.8-fold (log_2_ fold change of −1.92) in both gene loci (Fig. 4).

Down-regulation of bacterial surface antigen is likely a fitness strategy to cope with the constantly changing host environment. Evrard *et al*.^57^ showed that the capsule-deficient mutant of a *K*. *pneumoniae* clinical isolate could be more efficiently internalized by dendritic cells than its wild-type counterpart. It was suggested that the presence of a thick capsule at the surface of *K. pneumoniae* may interfere with binding and internalization by host cells. Similarly, in uropathogenic *E*. *coli*, King *et al*.^58^ reported that the capsule gene expression are spatially and temporally regulated during different phases of infection such that the unencapsulated cells in the population can preferentially adhere to and invade bladder epithelial cells, and only produce capsule once internalized for intracellular survival and spread. Our results further support these previous studies that expression of bacterial surface antigens are possibly regulated in response to interactions with host cells to evade host immune responses.

### Host responses to MDRO co-culture

**Figure 5:**
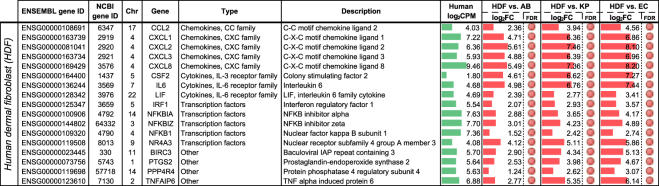
Human host genes were upregulated when HDF cells were co-cultured with MDROs. Differentially regulated genes shown with edgeR false discovery rate (FDR): <0.05 (red circle). Log_2_ fold change values of human gene expression was shown as red (up-regulated) bar graphs with reference to monocultures of HDF cells. Bar scale from 0 to 9. Averaged gene expression level across control and treatment experiments (log_2_ read counts per million, log_2_CPM) was shown as green bar graphs. Bar scale from 0 to 15.

With the same set of co-cultured assays in which MDROs were individually co-cultured with mammalian human fibroblast cells, we also measured human host gene responses. Analysis of HDF-derived human RNA-Seq reads revealed a set of 17 human genes that were significantly up-regulated across all three MDRO-fibroblast co-culture assays (Fig. 5). The up-regulated gene set included chemokines, cytokines, and transcription factors that were up-regulated from 2.36 (log_2_ fold change of 1.24) to 294-fold (log_2_ fold change of 8.2) across the three MDROs. Pathway and GO term enrichment analyses of the gene set were performed using DAVID Bioinformatics Resources (https://david.ncifcrf.gov)^59,60^. The top 5 enriched KEGG pathways and biological process GO terms were shown in Supplementary Table S8. The gene set was enriched in inflammatory and immune response functions. A total of 11 out of the 17 genes could be mapped to the Tumor necrosis factor (TNF) signaling pathway in KEGG (Supplementary Fig. S6)^61–63^. TNF is involved in the regulation of apoptosis, cell survival, and inflammation and immunity.

## Conclusion

In summary, we performed pairwise co-culture experiments of three selected high-priority military MDROs individually with each other, commensal and probiotic microbes, and cultured human cells. We monitored transcriptome responses of both the target and co-cultured pathogen/commensal species using RNA-Seq. Our goal was to delineate gene responses associated with virulence, survival mechanisms, and AMR in the MDROs with partners likely found in combat wound infections and potential probiotic scenarios rather than monoculture conditions.

Using this co-culturing approach, we observed differentially regulated gene responses associated with siderophore production, pili formation, and cell attachment. Our results suggested that a mixed microbial population may modulate the virulence and colonization of host cells. Future experiments are needed to determine the biological signals involved in the regulation of these important survival and virulence mechanisms of MDROs for effective drug targeting.

Our approach leveraged bacteria-bacteria and bacteria-host *in vitro* co-culture to observe differential transcriptional regulation using RNA-Seq analysis. This study highlights the diverse stimuli an organism may encounter in different environmental niches, and the complex gene regulation that takes place to achieve optimal survival and fitness. This type of analysis will be critical to understanding the interactions among the microbial pathogens, the host microbiome and the host environment, particularly in disease conditions. Our results provide foundational data and insight into the possibility of manipulating the local microbiome or avoiding ‘collateral-damage’ by minimizing exposure to overly broad-spectrum antibiotics or vancomycin, which kills skin commensals, when treating complicated polymicrobial wound, intra-abdominal, or respiratory infections.

## Methods

### Genome Sequencing

The genomes of the MDRO isolates were sequenced at JCVI by Illumina HiSeq (2 × 100 bp). Briefly, paired-end libraries were constructed for each sequencing technology from randomly nebulized genomic DNA in the 300–800 bp size range following manufacturer recommendations. Sequence reads were generated with a target average read depth of ~60-fold coverage.

### Draft Genome Assembly

To integrate the JCVI Illumina HiSeq data with data generated through various sequencing platforms and OpGen optical restriction maps generated by WRAIR, an Automated Gap Closure pipeline that combined *de novo* assembly followed by reference-guided gap closure to resolve short and uncomplicated gaps was implemented as described previously^50^.

### Genome annotation

Contigs were annotated for protein- and RNA-encoding features using the JCVI automated annotation pipeline essentially as described previously^50^. Genes conferring drug resistance were identified using the RGI (Resistance Gene Identifier, Version 2) tool in strict mode against the Comprehensive Antibiotic Resistance Database (CARD)^51–53^.

### Bacterial strains, growth conditions and bacterial co-culture experiments

MDROs *A*. *baumannii* MRSN 7339, *K*. *pneumoniae* MRSN 1319, and *E*. *hormaechei* MRSN 11489 were selected by WRAIR MRSN based on having the highest degree of clinical importance as described earlier in the results section and identified by standard automated biochemical analysis as described previously^64^. The selection criteria and antibiotic resistance profile of the three MDROs against 20 antibiotics were shown in Supplementary Tables S1 and S2, respectively. WRAIR MRSN provided purified genomic DNA and culture stocks of these MDROs. The Human Research Protection Office (HRPO) of the U.S. Army Medical Research and Material Command determined that this study was research not involving human subjects, as it did not “involve living individuals about whom an investigator conducting research obtains (1) data through intervention or interaction with the individual, or (2) identifiable private information, in accordance with 32 CFR 219.102(f).” Likewise, the JCVI IRB determined that the use of MDROs from MRSN was exempt from IRB review because the material provided was pre-existing and de-identified.

The commensal bacteria *Lactobacillus reuteri* SD2112 (ATCC 55730), *Staphylococcus epidermidis* SK135 (BEI HM-118), and *C*. *jeikeium* NCTC 11913 (ATCC 43734) were obtained from ATCC/BEI Resources, and therefore exempt from IRB review at JCVI since they are publicly available and de-identified from any human source. *A*. *baumannii*, *K*. *pneumoniae*, *E*. *hormaechei*, and *S*. *epidermidis* were grown in BHI broth (BD) to a concentration of 1 × 10^8^ CFU/ml in log phase growth. *C*. *jeikeium* and *L*. *reuteri* were grown to the same target concentrations in BHI supplemented with 1% (vol/vol) Tween-80 (Fisher) and MRS broth (BD), respectively. For co-culture assays, equal amounts of cells from the cultures were combined and shaken at 37 °C for 5 min at 200 RPM. 1 × 10^7^ CFU of each co-culture were plated onto a BHI agar plate in triplicates. Plates were incubated at 37 °C for 6 hours, at which point approximately 1 × 10^8^ CFU from each triplicate plate were combined and treated with RNAprotect bacteria reagent (Qiagen) according to the manufacturer’s instructions. Bacterial cells were spun down and the RNAprotect bacterial reagent removed after treatment. Cell pellets were stored at −80 °C until RNA extraction. Growth inhibition of *S*. *epidermidis* by *E*. *hormaechei* was analyzed by quantifying colony forming units (CFUs) on phenylethyl alcohol and Hektoen Enteric selective agar media^65,66^. The same experiment was performed using independent cultures on different days for biological replicates to control for reproducibility.

### Human dermal fibroblast growth conditions and co-culture experiments

Adult human dermal fibroblast (HDF) cells (Cat. no. C-013-5C) were obtained from Invitrogen and their experimental use in this study was found to be exempt from IRB review by the JCVI IRB because these cells are publicly available and de-identified. HDF cultures were set up to test multiplicity of infection (MOI) and incubation times for individual MDROs. The MOIs for all three MDROs with HDF cells was determined to be optimal at 100:1 (MDRO:HDF). The optimal duration of a bacterial-HDF co-culture assay was determined to be one hour. Prolonged co-culture times resulted in acidification of the cell culture media, which was avoided to eliminate interference with gene expression due to pH changes.

Multiple control experiments to determine retention of bacteria to HDF cells were performed and bacteria were observed via light microscopy to be adherent to the HDF cells, even after 3 rinses with PBS (data not shown). Cells (including MDRO bacteria and HDF cells) were isolated for RNA extraction from the co-culture assays after removing excess culture media so only adherent/close proximity bacterial cells were harvested. Cells were then treated with RNAprotect Cell Reagent (Qiagen) to immediately stabilize RNA transcripts before further processing.

### RNA isolation

RNA was extracted using the RNeasy Mini Kit (Qiagen). The manufacturer’s protocol was followed with additional steps as described below. Enzymatic lysis was performed on cell pellets resuspended in TE with 15 mg/ml lysozyme (Fisher) and 3 mg/ml Proteinase K (NEB) and incubated for 10 min at room temperature with vortexing for 10 s every 2 min. After enzymatic lysis, mechanical lysis was performed by adding buffer RLT with 1% vol/vol 2-mercaptoethanol and transferring the lysate to a Lysis Matrix B bead beating tube (MP Bio). Samples were homogenized for 45 s at 6.5 m/s on a FastPrep120 Cell Disrupter system. On-column DNAse treatment (Qiagen) was used according to the manufacturer’s protocol. Isolated RNA was treated with TURBO DNase (Ambion) following the manufacturer’s protocol.

### RNA-Seq library preparation for Illumina sequencing

Enrichment for mRNA was done using the RiboZero rRNA Removal Kit for bacteria (Epicentre). Libraries were constructed from 10 ng of mRNA using the NEBNext Ultra Directional RNA Library Prep Kit (NEB) following the manufacturer’s protocol. Libraries were normalized and pooled for sequencing using qPCR (Kapa Biosystems Library Quantification Kit). The resulting pooled libraries were sequenced on the Illumina HiSeq platform (2 × 100 bp).

### RNA-Seq data analysis

Illumina sequencing reads in FASTQ format were analyzed using CLC Genomics Workbench version 6.5 (http://www.clcbio.com) after removing low quality reads (CLC quality score limit = 0.05, maximum of 2 ambiguities), reads less than 50 bp, and adaptors. To remove rRNA reads, a set of known 4,692 distinct rRNA sequences were collected from the SILVA database (http://www.arb-silva.de/) for all five species of bacteria used in the co-culture assays. All reads mapped to the known rRNA sequences were discarded. BWA^67^ was used for the mapping allowing up to three mismatches. Reference files for RNA-Seq analysis were prepared from Genbank files imported into CLC. For each pair of control and co-culture experiment, the expected genomes were selected and reads were mapped using the following settings: maximum number of mismatches = 2, minimum length fraction = 0.8, minimum similarity fraction = 0.98, maximum number of hits for a read = 10, minimum and maximum distances for paired reads = 1,1000 and counting scheme of “include broken pairs”. All reads mapped to each gene were used as raw read count to determine up or down-regulated genes using the Bioconductor package edgeR^68^. Genes with at least 1 CPM in at least 2 samples were included for the differential expression analysis. Differentially expressed genes were determined using an exact test implemented for negative-binomially distributed counts with estimated genewise dispersion values using edgeR. The RNA-Seq gene expression for the three MDROs *A*. *baumannii*, *K*. *pneumoniae*, and *E*. *hormaechei*, together with curated virulence gene annotation and AMR annotation are shown in Supplementary Tables S4–S6.

### Reverse Transcription and qRT-PCR

Reverse transcription (RT) was performed to generate cDNA from two biological replicates to be used in qRT-PCR for the validation of RNA-Seq data. RNA concentrations were determined with an Agilent 2100 Bioanalyzer and 1.5 μg of total RNA was used in each reaction. RNA was incubated with 6 μg of random hexamers (Invitrogen) and 40 U of RNaseOUT (Invitrogen) in 18.5 μl at 70 °C for 10 min, followed by snap cooling on ice. The reaction was set up with 1 × First Strand Buffer, 10 mM DTT, 0.5 mM dNTPs (Invitrogen), and 400 U SuperScript® III Reverse Transcriptase (Invitrogen) in 30 μl. The reaction was incubated in a 42 °C water bath for 16 h. The reaction was stopped and RNA was hydrolyzed with 0.1 M EDTA and 0.2 M NaOH at 65 °C for 12 min. Tris-HCl (pH 7.0) was added to neutralize the pH. Purification of cDNA was achieved by using QIAquick PCR Purification kit (Qiagen) following the manufacturer’s protocol and quantitated by fluorometric assays using SYBR Gold (Invitrogen).

qRT-PCRs were performed with 10 ng of template cDNA in 10 μl triplicate reactions with SYBR Green Master Mix (Roche) in 384-well plates on the LightCycler480 qPCR instrument (Roche). Cycling conditions were 95 °C for 10 min followed by 50 cycles of 95 °C for 20 s, 58 °C for 30 s, and 72 °C for 15 s. A melting curve analysis was performed after all cycles were completed to verify the Tm of amplified products. No-RT controls and qPCR control reactions were performed as well. To determine the C_P_ value (crossing point-PCR-cycle or threshold cycle) for each reaction, the ‘Fit Point Method’ was performed in the LightCycler480 software 1.5.0 (Roche Diagnostics). Efficiency for each primer was optimized by adjusting primer concentration and was determined for the reaction conditions. Efficiency and C_P_ values were used to calculate the gene expression with normalization to *gyrB*. Relative expression ratios were determined by comparing co-culture samples with monoculture samples using the method described previously^69^. Primer sequences and concentrations used are provided in Supplementary Table S9.

### Data availability

Genome assembly and annotation for the three selected MDRO isolates are available under NCBI bioprojects: PRJNA223624, PRJNA223615 and PRJNA223617. RNA-Seq datasets for the co-culture assays are available at the NCBI SRA under bioprojects: PRJNA292770, PRJNA292776, PRJNA292777, PRJNA294773, PRJNA294780, PRJNA294781 and PRJNA314266.

## Electronic supplementary material

Supplementary Tables

Supplementary Information
