# Supplementary material for: Multidrug resistant pathogens respond differently to the presence of co-pathogen, commensal, probiotic and host cells
Source: Sci Rep. 2018 Jun 5;8:8656. doi: 10.1038/s41598-018-26738-1 (PMC5988826; doi:10.1038/s41598-018-26738-1)

Multidrug resistant pathogens respond differently to the presence of co-pathogens, commensals, probiotics and host cells

Agnes P. Chan[1†*], Yongwook Choi[1†], Lauren M. Brinkac[1], Radha Krishnakumar[1], Jessica DePew[1], Maria Kim[1], Mary K. Hinkle[2], Emil P. Lesho[2,3^], and Derrick E. Fouts[1*]

[1]J. Craig Venter Institute (JCVI), 9605 Medical Center Drive, Suite 150, Rockville, MD 20850, United States

[2]Multidrug-resistant organism Repository and Surveillance Network, Bacterial Diseases Branch, Walter Reed Army Institute of Research, Silver Spring, MD 20910, United States

[3] Infectious Diseases Unit, Rochester Regional Health, Rochester, NY, 14621

^ Current affiliation

*corresponding authors: dfouts@jcvi.org, achan@jcvi.org

[†]these authors contributed equally to this work

Figure S1. Growth of *S. epidermidis* and *E. hormaechei* in co-culture and mono-culture. *S. epidermidis* colonies were selected for by growth on phenylethyl-alcohol agar from co-culture with *E. hormaechei* (blue), or mono-culture (red). *E. hormaechei* colonies were selected for by growth on Hektoen Enteric agar from co-culture with *S. epidermidis* (green), or mono-culture (purple). Co-culture datapoints are denoted with diamonds while monoculture datapoints with Xs.

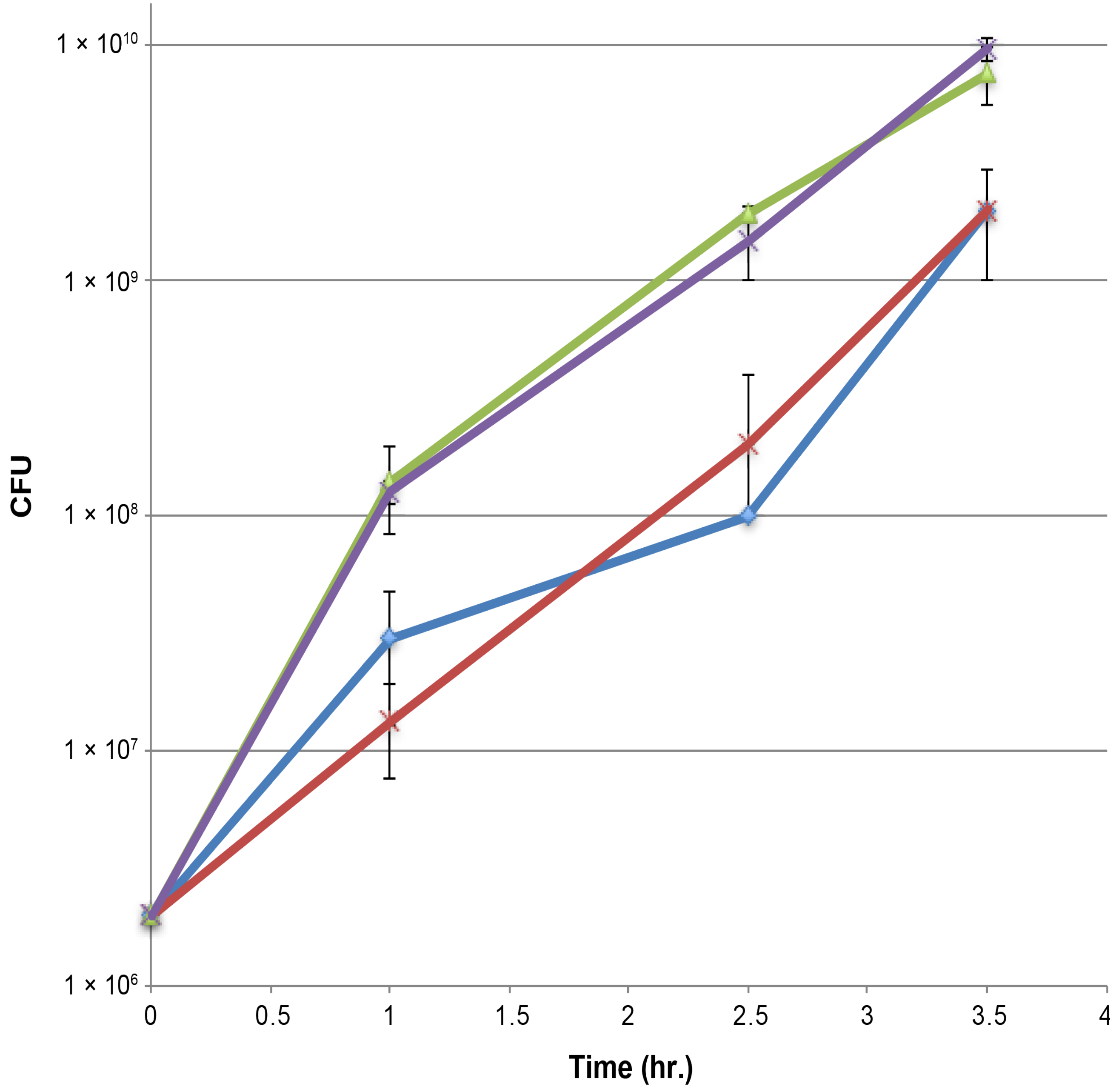

Figure S2. Top gene responses of *A. baumannii* when co-cultured independently with *E. hormaechei* or *K. pneumoniae.*

Genes with at least 4-fold up or down expression and FDR above 5% in at least one co-culture are shown.

NCBI annotation			Symbol	AB vs. EH		AB vs. KP	
Locus tag	Product	Protein id		log_2FC	FDR	log_2FC	FDR
T634_RS01410	aldehyde dehydrogenase	WP_001269058.1		3.60	●	3.62	●
T634_RS01395	alcohol dehydrogenase	WP_000874704.1		3.56	●	3.60	●
T634_RS06340	lipoyl synthase	WP_000364558.1	lipA	3.41	●	3.57	●
T634_RS10775	gamma-aminobutyraldehyde dehydrogenase	WP_001140777.1		3.24	●	2.40	●
T634_RS06355	diaminohydroxyphosphoribosylaminopyrimidine deaminase	WP_001289728.1		2.98	●	2.90	●
T634_RS06345	ABC transporter substrate-binding protein	WP_001177517.1	acoA	2.89	●	3.04	●
T634_RS06350	alpha-ketoacid dehydrogenase subunit beta	WP_001133051.1	acoB	2.85	●	2.90	●
T634_RS17975	acetate kinase	WP_000236000.1	ackA	2.83	●	2.78	●
T634_RS08500	hypothetical protein	WP_001991318.1		2.83	●	0.12	
T634_RS06360	dihydrolipoyl dehydrogenase	WP_001287542.1	lpdA	2.76	●	2.75	●
T634_RS10780	polyketide cyclase	WP_001041177.1		2.72	●	1.92	●
T634_RS18375	NAD(P)(+) transhydrogenase (Re/Si-specific) subunit alpha	WP_001165468.1		2.72	●	2.70	●
T634_RS18380	NAD(P) transhydrogenase subunit alpha	WP_000230434.1		2.68	●	2.65	●
T634_RS08960	isocitrate lyase	WP_000214787.1		2.61	●	2.73	●
T634_RS18385	NAD(P) transhydrogenase subunit beta	WP_000394405.1	pntB	2.55	●	2.50	●
T634_RS17970	phosphate acetyltransferase	WP_001092295.1	pta	2.51	●	2.63	●
T634_RS08515	heme oxygenase	WP_001089060.1		2.49	●	-0.22	
T634_RS08505	DUF560 domain-containing protein	WP_000831634.1		2.41	●	-0.21	
T634_RS08495	hypothetical protein	WP_004715426.1		2.39	●	-0.45	
T634_RS08510	energy transducer TonB	WP_000837763.1		2.07	●	-0.56	
T634_RS16990	cation acetate symporter	WP_000871885.1	actP	2.01	●	2.06	●
T634_RS16995	DUF485 domain-containing protein	WP_000372818.1		1.98	●	2.09	●
T634_RS08910	esterase	WP_000697203.1		1.97	●	2.05	●
T634_RS13075	sulfate ABC transporter substrate-binding protein	WP_001212600.1	sbp	1.66	●	2.28	●
T634_RS05115	taurine ABC transporter permease	WP_001092656.1		1.66	●	2.03	●
T634_RS04825	hypothetical protein	WP_000108365.1		-1.27		-2.27	●
T634_RS14335	acyl-CoA dehydrogenase	WP_001160766.1		-1.96	●	-2.08	●
T634_RS14345	enoyl-CoA hydratase/isomerase family protein	WP_001091033.1		-2.02	●	-2.11	●
T634_RS14340	enoyl-CoA hydratase	WP_001198691.1		-2.03	●	-1.97	●
T634_RS14355	N-acylhomoserine lactone synthase	WP_001020940.1		-2.09	●	-2.09	●
T634_RS03745	DUF2280 domain-containing protein	WP_000113266.1		-2.13	●	-2.14	●
T634_RS10710	EamA family transporter	WP_001018285.1		-2.30	●	-1.05	●
T634_RS14350	MFS transporter	WP_001193286.1		-2.33	●	-2.53	●
T634_RS14320	methylmalonate-semialdehyde dehydrogenase (CoA acylating)	WP_001092394.1	mmsA	-2.34	●	-2.44	●
T634_RS14725	penicillin G amidase	WP_000935120.1		-2.36	●	-2.34	●
T634_RS14720	3-oxoadipate enol-lactonase	WP_001138966.1	pcaD	-2.37	●	-2.37	●
T634_RS14325	3-hydroxyisobutyrate dehydrogenase	WP_001017291.1	mmsB	-2.43	●	-2.77	●
T634_RS14330	AMP-binding protein	WP_000969576.1		-2.44	●	-2.82	●
T634_RS01875	dicarboxylate/amino acid:cation symporter	WP_000347180.1	dctA	-2.51	●	-2.31	●
T634_RS14700	catechol 1,2-dioxygenase	WP_001081618.1	catA	-2.89	●	-2.77	●
T634_RS14805	outer membrane porin, OprD family	WP_000421436.1		-3.05	●	-2.90	●
T634_RS14690	muconate cycloisomerase	WP_001986121.1	catB	-3.28	●	-2.91	●
T634_RS14695	muconolactone delta-isomerase	WP_000897235.1	catC	-3.32	●	-3.28	●
T634_RS14800	MFS transporter	WP_001076622.1		-3.33	●	-3.13	●

Figure S3. Top gene responses of *K. pneumoniae* when co-cultured independently with *A. baumannii* or *E. hormaechei.*

Genes with at least 4-fold up or down expression and FDR above 5% in at least one co-culture are shown.

NCBI annotation			Symbol	Virulence annotation (This study)			KP vs. AB		KP vs. EH	
Locus tag	Product	Protein id		Category	Description	Other	log_2FC	FDR	log_2FC	FDR
T643_RS07920	TonB-dependent receptor	WP_032434976.1					5.49		-0.02	
T643_RS07935	iron ABC transporter permease	WP_004174663.1					5.27		-0.46	
T643_RS07930	hemin ABC transporter substrate-binding protein	WP_004181072.1	hmuT				5.18		-1.01	
T643_RS07925	hemin-degrading factor	WP_032434978.1	hmuS				5.10		-0.92	
T643_RS07800	hydrogenase nickel incorporation protein HypA	WP_032412123.1	hypA				4.80		3.01	
T643_RS07795	formate hydrogenlyase regulatory protein HycA	WP_002914925.1	hycA				4.36		-4.56	
T643_RS12575	phosphatase	WP_009483944.1					4.34		0.22	
T643_RS07940	heme ABC transporter ATP-binding protein	WP_004149516.1	hmuV				3.87		-0.23	
T643_RS12570	DUF1348 domain-containing protein	WP_016529674.1					3.67		-0.10	
T643_RS07915	hypothetical protein	WP_002915045.1					3.34		0.49	
T643_RS00915	NarK family nitrate/nitrite MFS transporter	WP_004175568.1	narK				3.29		-0.42	
T643_RS07500	class 1b ribonucleoside-diphosphate reductase su	WP_032434956.1	nrdF				2.93		-0.86	
T643_RS07490	class Ib ribonucleoside-diphosphate reductase as	WP_002914321.1	nrdI				2.80		-1.05	
T643_RS07485	NrdH-redoxin	WP_002914320.1	nrdH				2.70		-1.11	
T643_RS07495	class 1b ribonucleoside-diphosphate reductase su	WP_032434955.1	nrdE				2.69		-1.02	
T643_RS12565	alkylhydroperoxidase	WP_004145017.1					2.35		-0.25	
T643_RS26565	hypothetical protein	WP_047682061.1					2.30		2.64	
T643_RS26185	catecholate siderophore receptor Fiu	WP_032434366.1	fiu				2.28		-1.08	
T643_RS24620	antirestriction protein ArdA	WP_015065510.1					2.21		2.15	
T643_RS07790	formate hydrogenlyase subunit 2	WP_004151047.1	hyfA				2.19		-0.49	
T643_RS26320	YmiA family putative membrane protein	WP_002901776.1					2.17		0.83	
T643_RS07910	acetolactate synthase	WP_032434974.1					2.14		-0.09	
T643_RS01280	bifunctional threonine ammonia-lyase/L-serine am	WP_002910762.1	tdcB				2.10		-1.71	
T643_RS03365	periplasmic protein	WP_047680786.1					2.02		-1.74	
T643_RS21130	catecholate siderophore receptor CirA	WP_002912926.1	cirA				1.96		-2.36	
T643_RS03815	isochorismate synthase EntC	WP_004176916.1	entC	Siderophores	Enterobactin	entC	1.36		-2.54	
T643_RS03820	2,3-dihydroxybenzoate-AMP ligase	WP_012068426.1		Siderophores	Enterobactin	entE	1.21		-2.77	
T643_RS23530	head completion/stabilization protein	WP_002896151.1					1.07		-2.57	
T643_RS03835	proofreading thioesterase EntH	WP_002893892.1					0.99		-2.35	
T643_RS03830	2,3-dihydro-2,3-dihydroxybenzoate dehydrogenas	WP_023159213.1		Siderophores	Enterobactin	entA	0.99		-2.66	
T643_RS03825	isochorismatase	WP_021313366.1	entB	Siderophores	Enterobactin	entB	0.90		-2.96	
T643_RS03775	enterochelin esterase	WP_040169968.1	fes	Siderophores	Enterobactin	fes	0.90		-2.47	
T643_RS03780	invasin	WP_004178967.1		Siderophores	Enterobactin	mbtH	0.87		-2.63	
T643_RS15710	hypothetical protein	WP_002906780.1					0.86		-2.01	
T643_RS03765	enterobactin synthase subunit EntD	WP_032434174.1		Siderophores	Enterobactin	entD	0.80		-2.31	
T643_RS19350	citrate/sodium symporter CitN	WP_023282163.1	citS				0.70		-2.37	
T643_RS03770	TonB-dependent siderophore receptor	WP_004210903.1	fepA_1	Siderophores	Enterobactin	fepA	0.70		-2.73	
T643_RS03805	enterobactin transporter EntS	WP_004147525.1		Siderophores	Enterobactin	entS	0.62		-2.77	
T643_RS19355	[citrate (pro-3S)-lyase] ligase	WP_032435569.1	citC				0.59		-2.06	
T643_RS23550	hypothetical protein	WP_047681893.1					0.55		-2.39	
T643_RS03785	enterobactin synthase subunit F	WP_047680809.1	entF	Siderophores	Enterobactin	entF	0.45		-2.70	
T643_RS03800	iron-enterobactin transporter membrane protein	WP_047680815.1	fepD	Siderophores	Enterobactin	fepD	0.42		-2.23	
T643_RS19360	citrate lyase ACP	WP_004151373.1	citD				0.40		-2.62	
T643_RS19365	citrate lyase subunit beta	WP_004151372.1	citE				0.38		-2.55	
T643_RS01335	TonB-dependent siderophore receptor	WP_032434771.1					0.19		-2.63	
T643_RS19370	citrate lyase subunit alpha	WP_032433050.1	citF				0.17		-2.54	
T643_RS19335	sodium ion-translocating decarboxylase subunit b	WP_023316443.1	oadB				-0.14		-2.64	
T643_RS16995	ribose ABC transporter ATP-binding protein	WP_002885156.1					-0.21		-2.03	
T643_RS19260	Na+/H+ antiporter NhaA	WP_032433058.1	nhaA				-0.22		2.00	
T643_RS07880	manganese transporter	WP_009485519.1					-0.33		-2.03	
T643_RS07885	metal ABC transporter permease	WP_004145794.1	sitC				-0.35		-2.01	
T643_RS18585	ethanolamine utilization protein EutN	WP_004182807.1					-0.84		-6.63	
T643_RS19340	oxaloacetate decarboxylase subunit alpha	WP_023282162.1	oadA				-0.86		-3.56	
T643_RS19345	oxaloacetate decarboxylase subunit gamma	WP_011977638.1					-0.94		-3.85	
T643_RS00795	oligopeptide ABC transporter permease OppC	WP_004145396.1	oppC				-2.04		-0.32	
T643_RS11485	4-aminobutyrate--2-oxoglutarate transaminase	WP_002920814.1	gabT_2				-2.04		0.29	
T643_RS09285	short chain dehydrogenase	WP_002916526.1					-2.07		-0.58	
T643_RS22620	phosphoribosylformylglycinamidine synthase	WP_031588782.1	purL				-2.10		-0.81	
T643_RS19850	bifunctional aconitate hydratase 2/2-methylisocitra	WP_074421634.1	acnB				-2.12		-0.82	
T643_RS11865	dipeptide ABC transporter permease DppC	WP_004145207.1	dppC				-2.12		-0.60	
T643_RS12885	phosphoribosylamine--glycine ligase	WP_002884357.1	purD				-2.13		-0.86	
T643_RS00390	3-(3-hydroxyphenyl)propionate hydroxylase	WP_032434708.1	mhpA				-2.15		-0.81	
T643_RS13260	CoA transferase subunit B	WP_002908192.1					-2.20		-1.39	
T643_RS09290	alpha/beta hydrolase	WP_004174434.1					-2.24		-1.16	
T643_RS01665	formate-dependent phosphoribosylglycinamide fo	WP_004151450.1	purT				-2.36		-0.69	
T643_RS12890	bifunctional phosphoribosylaminoimidazolecarbox	WP_004178254.1	purH				-2.48		-0.97	
T643_RS15470	VOC family protein	WP_002906451.1					-2.81		-0.84	
T643_RS14390	ABC transporter	WP_073553711.1					-3.04		-1.90	

Figure S4. Top gene responses of *E. hormaechei* when co-cultured independently with *A. baumannii* or *K. pneumoniae.*

Genes with at least 4-fold up or down expression and FDR above 5% in at least one co-culture are shown.

NCBI annotation			Symbol	Virulence annotation (This study)			EH vs. AB		EH vs. KP	
Locus tag	Product	Protein id		Category	Description	Other	log_2FC	FDR	log_2FC	FDR
T636_RS04035	ligand-gated channel protein	WP_045350507.1					5.01	🔴	3.09	🔴
T636_RS04030	Hemin uptake protein	WP_015570723.1					4.39	🔴	3.52	🔴
T636_RS04045	hemin ABC transporter substrate-binding protein	WP_022650910.1	hmuT				4.36	🔴	2.63	🟡
T636_RS11800	2,3-dihydroxybenzoate-AMP ligase	WP_047654086.1		Siderophores	Enterobactin	entE	4.30	🔴	2.70	🔴
T636_RS04040	hemin-degrading factor	WP_022650911.1	hmuS				4.22	🔴	2.69	🔴
T636_RS04050	iron ABC transporter permease	WP_045336621.1					4.20	🔴	2.38	🔴
T636_RS11805	isochorismate synthase EntC	WP_023324292.1	entC	Siderophores	Enterobactin	entC	4.18	🔴	2.56	🟡
T636_RS07205	ligand-gated channel protein	WP_045896695.1					4.12	🔴	2.23	🟡
T636_RS11795	isochorismatase	WP_017382510.1	entB	Siderophores	Enterobactin	entB	3.79	🔴	2.27	🟡
T636_RS11790	2,3-dihydro-2,3-dihydroxybenzoate dehydrogenase	WP_059585462.1		Siderophores	Enterobactin	entA	3.38	🔴	2.10	
T636_RS11845	enterochelin esterase	WP_045350568.1	fes	Siderophores	Enterobactin	fes	3.30	🔴	2.44	🔴
T636_RS11840	hypothetical protein	WP_003858806.1		Siderophores	Enterobactin	mbtH	3.26	🔴	2.34	🔴
T636_RS11785	proofreading thioesterase EntH	WP_015571173.1					3.21	🔴	1.77	
T636_RS11850	TonB-dependent siderophore receptor	WP_047654088.1	fepA	Siderophores	Enterobactin	fepA	3.17	🔴	2.22	🔴
T636_RS11835	enterobactin synthase subunit F	WP_047654087.1	entF	Siderophores	Enterobactin	entF	3.08	🔴	2.02	🔴
T636_RS19095	hypothetical protein	WP_047654473.1					2.63	🔴	1.77	🔴
T636_RS12020	enterochelin esterase	WP_045332430.1					2.34	🔴	1.20	
T636_RS01165	aerobactin synthase IucA	WP_047653418.1	iucA	Siderophores	Aerobactin	iucA	2.27	🔴	1.44	🔴
T636_RS01185	TonB-dependent siderophore receptor	WP_023324924.1		Siderophores	Aerobactin	iutA	2.11	🔴	1.35	
T636_RS04055	heme ABC transporter ATP-binding protein	WP_032658174.1	hmuV				2.06	🔴	0.93	
T636_RS01175	IucA/IucC family siderophore biosynthesis protein	WP_023324922.1		Siderophores	Aerobactin	iucC	2.06	🔴	1.52	🔴
T636_RS01170	N-acetyltransferase	WP_023314431.1		Siderophores	Aerobactin	iucB	2.06	🔴	1.39	
T636_RS18005	nitrite reductase small subunit NirD	WP_015571429.1	nirD				2.05	🔴	0.43	
T636_RS01180	lysine 6-monooxygenase	WP_047653419.1	iucD	Siderophores	Aerobactin	iucD	2.04	🔴	1.38	🟡
T636_RS15175	hypothetical protein	WP_003862457.1					-2.13	🔴	-0.20	

Figure S5. Validation of Differentially Expressed genes. Lines indicated logFC (base 2) expression from edgeR analysis of RNA-Seq data, and bars indicate logFC (base 2) expression as measured by qPCR. Blue bars are *K. pneumoniae* genes, and gray bars are *A. baumannii* genes. x-axis labels showed: organism, locus tag, and gene name, followed by the co-culture assay in brackets. Key: *K. pneumoniae* (KP), *A. baumannii* (AB), *E. hormaechei* (EH), *L. reuteri* (LR), *S. epidermidis* (SE) and *C. jeikeium* (CJ).

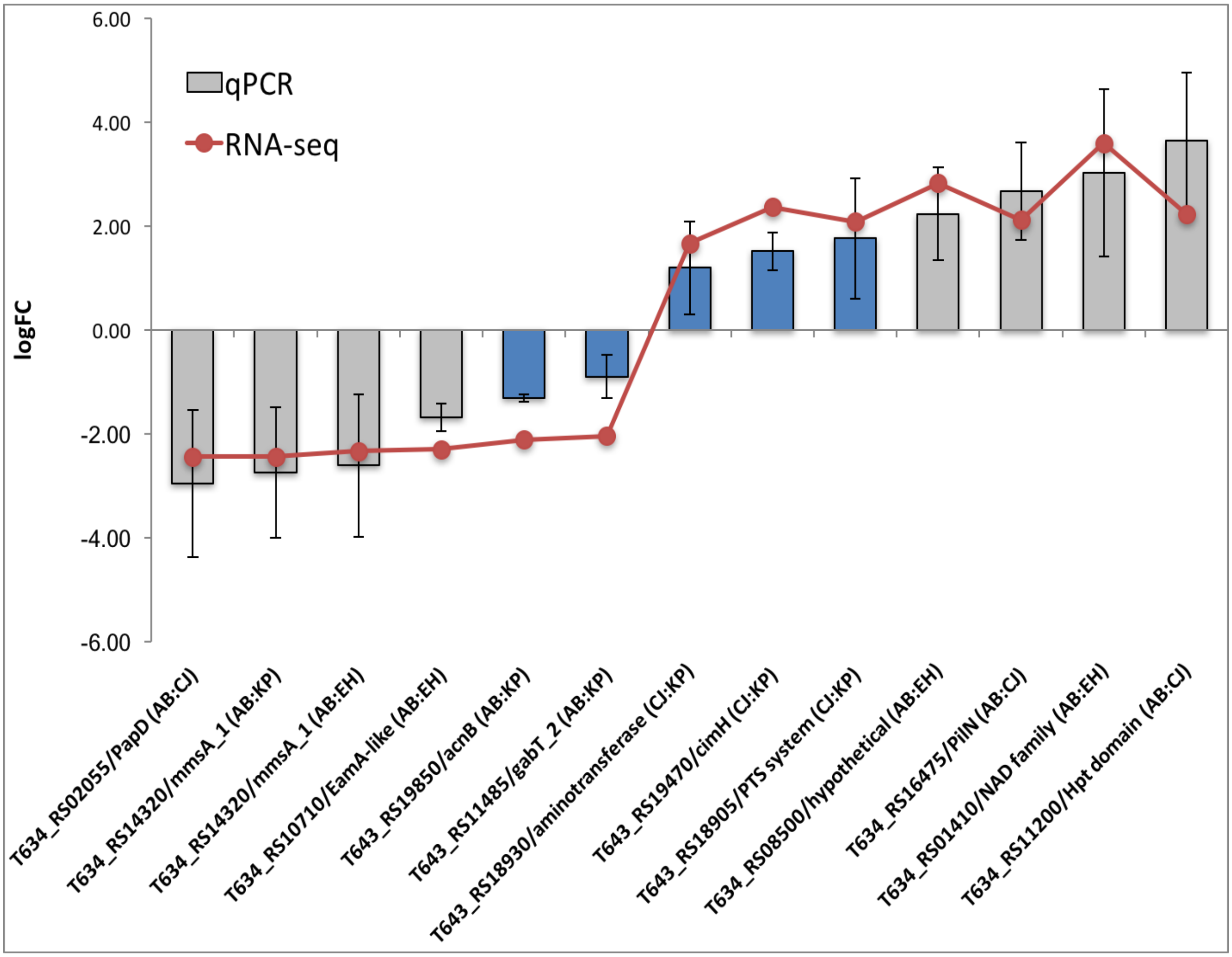

Figure S6. Mapping of upregulated human host genes to the TNF signaling pathway (KEGG) shared across MDRO and HDF co-culture assays.

A total of 11 out of 17 up-regulated human genes (red star) belonged to the TNF signaling pathway as shown in the KEGG pathway image (KEGG, http://www.genome.jp/kegg-bin/show_pathway?hsa04668).

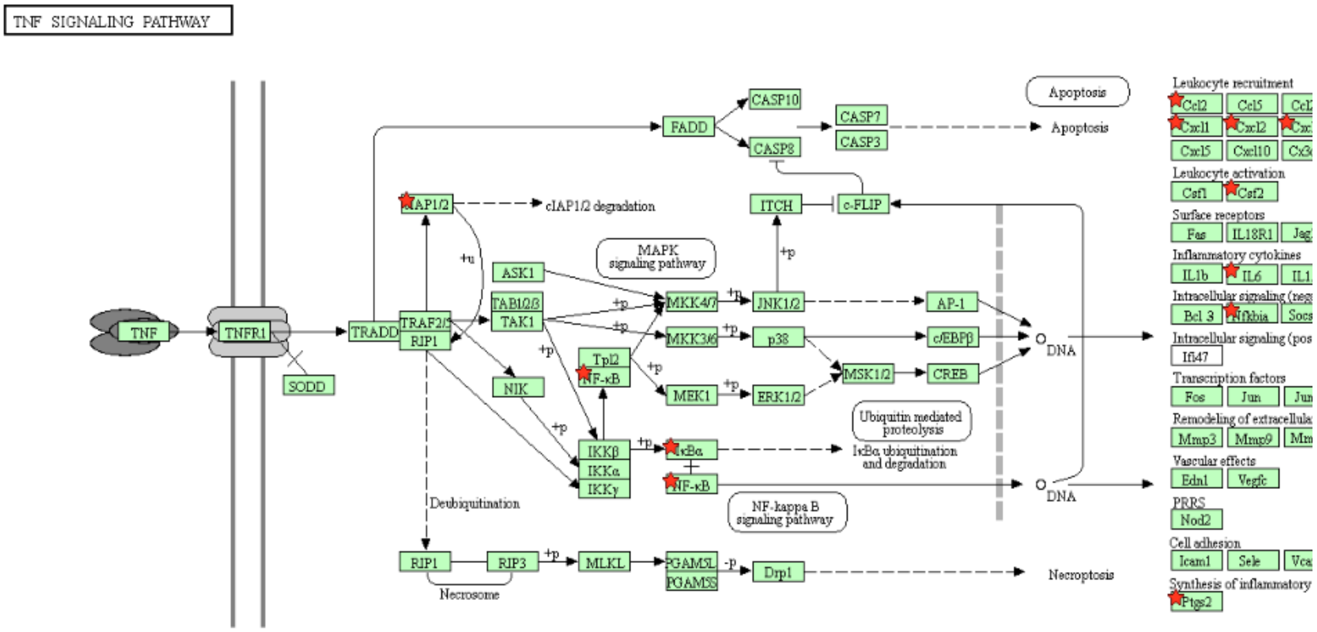

Supplement: Supplementary file 2 — Supplementary Information [file 41598_2018_26738_MOESM2_ESM.pdf]
